# Sleep Duration and Executive Function in Adults

**DOI:** 10.1007/s11910-023-01309-8

**Published:** 2023-11-14

**Authors:** Aayushi Sen, Xin You Tai

**Affiliations:** 1 Nuffield Department of Clinical Neurosciences, University of Oxford, Oxford, UK; 2 Division of Clinical Neurology, John Radcliffe Hospital, Oxford University Hospitals Trust, Level 6 West Wing, Oxford, UK

**Keywords:** Sleep duration, Sleep quality, Executive function, Dementia

## Abstract

**Purpose of Review:**

To review the literature examining the relationship between sleep and cognition, specifically examining the sub-domain of executive function. We explore the impact of sleep deprivation and the important question of how much sleep is required for optimal cognitive performance. We consider how other sleep metrics, such as sleep quality, may be a more meaningful measure of sleep. We then discuss the putative mechanisms between sleep and cognition followed by their contribution to developing dementia.

**Recent Findings:**

Sleep duration and executive function display a quadratic relationship. This suggests an optimal amount of sleep is required for daily cognitive processes. Poor sleep efficiency and sleep fragmentation are linked with poorer executive function and increased risk of dementia during follow-up. Sleep quality may therefore be more important than absolute duration. Biological mechanisms which may underpin the relationship between sleep and cognition include brain structural and functional changes as well as disruption of the glymphatic system.

**Summary:**

Sleep is an important modifiable lifestyle factor to improve daily cognition and, possibly, reduce the risk of developing dementia. The impact of optimal sleep duration and sleep quality may have important implications for every ageing individual.

## Introduction 

Sleep is an integral part of human life and is linked to optimal performance across a broad range of physiological and psychological functions [1–4]. The relationship between sleep and executive function is an area of intense interest as optimising sleep may be one avenue to improve cognition as we grow older. Executive function, a critical cognitive domain for day-to-day living, has been closely linked to sleep patterns as we grow older. For instance, sleep deprivation is associated with increased frequency of mistakes by shift workers [5] and increased reliance on habits rather than goal-directed decisions that require executive control [6]. Despite a growing body of literature, the exact nature of the relationship between sleep and cognition remains unclear. Importantly, what is the optimal amount of sleep required for cognitive functioning? Does this change as we age? These are not straightforward questions, as studies have highlighted different sleep lengths as detrimental or beneficial for cognitive performance. Additionally, how strong is the causal relationship between sleep and cognition? This is crucial if sleep is to be considered a key modifiable lifestyle factor to optimise cognition and mitigate risk of certain brain disorders, especially dementia.

This article offers an overview of the current literature examining sleep duration and executive function in mid-to-late life and will explore key issues including potential underlying mechanisms, the link with brain structural health, and potential contribution to developing dementia.

## The Importance of Executive Function

Executive function is the orchestration of goal-oriented processes that include attention, problem solving, planning, and working memory. This includes the ability to hold information in your short-term memory, manipulate that information, and decide which part of the information is important for the task at hand. Executive functioning is particularly developed in humans compared to other animals, and is important for performing everyday tasks ranging from getting dressed, following a recipe, driving a car to more complex problems [7]. During adulthood, executive function declines with age along with several other cognitive domains [8–10]. Furthermore, executive function is commonly affected across a wide range of neurological and psychological disorders such as dementia (particularly fronto-temporal dementia), stroke, and head trauma [11–14]. It is therefore critical to understand modifiable factors, such as sleep, that could potentially optimise executive function.

### How is Executive Function Tested?

Executive function is tested in several ways. Common measures include goal-oriented tasks like the trail-making test [15], digit-symbol substitution test (DSST) [16], Wisconsin card sorting test [17], or the Stroop test [18, 19]. Such tasks require a combination of attention, online processing of information, and cognitive effort. Some tasks will engage cognitive control, whereby participants have to decide when to act (‘GO’) but also when not to act (‘NO-GO’), while others may require a participant to place themselves in the mind of another person (theory of mind). More general questionnaires of cognition, such as the Mini Mental State Examination (MMSE) [20] and Montreal Cognitive Assessment (MoCA) [21], may have subcomponents of executive function. These can be useful as scalable tests of cognitive ability for large-scale studies but do not assess executive function with the detail of dedicated tasks. By contrast, there are specific cognitive tasks of executive function created to answer specific questions [22, 23], but these may be hard to incorporate into larger studies. Box 1 demonstrates 3 tests you can try yourself.
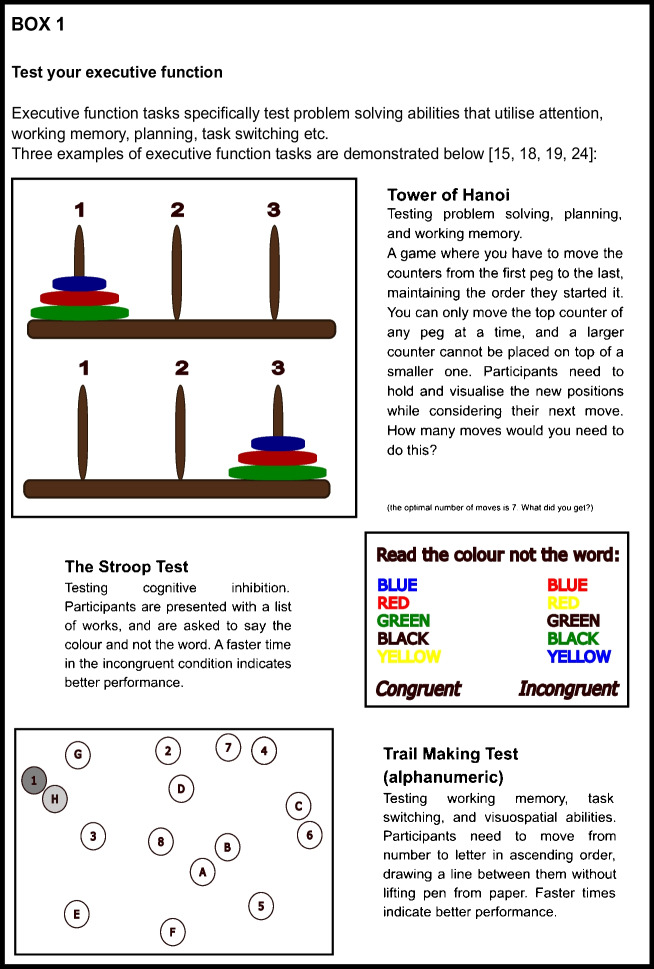


## Function

Sleep deprivation is common, with 11.8% of respondents reporting less than 5 h sleep on average in a large US survey [25]. Deficits in motor performance due to sleep deprivation are equivalent to blood alcohol content of 0.05–0.1%, which is comparable to the legal driving limit of 0.08% [26] in England and the USA. A single night of sleep deprivation has been shown to affect several components of executive function such as sustained attention, reaction time, and working memory, as well as other cognitive domains of consolidation of episodic and procedural memory [27].

Sleep deprivation experiments can involve keeping participants awake for an extended period of time (usually over 24 h), or restricting sleep to only a few hours over multiple days. Cognition is tested before, during, and after sleep deprivation periods. Tasks requiring sustained attention show worse performance over 28 h of sleep deprivation [28]; with more pronounced effects, the more mundane the task is [29]. Creative thought processes are affected more than rule-based processes [30], and people revert to habitual actions rather than goal-directed actions for the task at hand [6].

The real-life impact of sleep deprivation is exemplified in studies of risk aversion, with several prominent studies examining occupations such as the military. Sleep-deprived individuals have impaired risk perception, where they performed worse in a simulated balloon overinflation experiment after 36 h of staying awake. Doing well on this task requires participants to pay attention to the balloon; contextualise it with previous inflation attempts; assess, in real time, the odds of the balloon popping; and inhibit the urge to score higher with a bigger balloon. Interestingly, poor performance in sleep-deprived participants corresponded to altered brain measures of network connectivity, compared to when the same individual was not sleep deprived [31]. This intra-subject analysis suggests that sleep deprivation may alter the way information is communicated through the brain. A meta-analysis, which pooled large amounts of data from multiple studies, involving 1341 sleep restricted military participants identified a significant negative effect on reaction times, processing speed, accuracy, and moral decision making [32].

In addition to executive function, sleep has been shown to be important for memory consolidation. Even short naps (as little as 6 min) can improve memory retention, with longer durations being particularly useful for procedural memory. Behaviourally relevant memories are favoured in sleep-dependent consolidation [33]. Therefore, unsurprisingly, sleep deprivation can negatively affect the consolidation of new memories, especially episodic [33, 34] and procedural memory [35–37]. Importantly, sleep recovery (being able to sleep a ‘normal’ amount) over the course of a week can lead to improved performance in previously sleep-deprived individuals, back to the level of controls [36].

Sleep extension (sleeping longer than normal) in the short term can reduce the effect of sleep deprivation on sustained attention tasks [38] and memory [36]. There are also interesting studies investigating factors that affect resilience against sleep deprivation. Older adults showed worse performance, compared to younger adults, following sleep deprivation in multiple cognitive tasks, including those testing vigilance and reaction times [39]. By contrast, older adults did not get a benefit from interval sleep after a motor-sequence learning task, unlike their younger counterparts [40], indicating unequal reliance on sleep for different ages and types of memory consolidation. This effect has not been observed in related non-motor learning paradigms [41].

Therefore, there is robust experimental and real-life evidence that acute periods of sleep deprivation can detrimentally affect cognition. A different question remains—what is the optimal daily duration of sleep to maximise our cognitive functioning? This is relevant to the daily lifestyle habits of all ageing individuals, and may provide insight into those with the worst cognitive functioning, such as in dementia.

## Long-Term Effect of Sleep: Both Short and Long Sleep Durations are Associated with Poorer Executive Function

Numerous studies examine the relationship between average sleep duration and executive function. A common way to probe this question has been to ask participants to self-report the average hours of sleep they had recently, and combine it with cognitive tests of executive function administered at a time point within the study. Using this approach, important findings have emerged.

### Short and Long Sleep Durations are Related to Worse Executive Function in Cross-sectional Studies

Earlier cross-sectional studies have associated worse executive function with either short or long extremes of sleep duration [42–48], while more recent studies tend to link both short and long sleep durations with poor executive function [49–58]. There may be several reasons for these mixed findings.

Firstly, studies tend to use a variety of methods to test cognition. One study of 3212 individuals aged over 60 demonstrated a worse MMSE score for every hour over 7 h of sleep per day in a linear analysis, but no difference in short sleepers [42]. Similar findings were also identified in a study which accounted for sleep-disordered breathing, which is often cited as a missed confounder in long sleep duration and cognitive impairment [50]. A linearly worsening trend at longer sleep durations was also seen in a study of executive function (testing DSST). This trend persisted after adjusting for sex, age, education, and BMI, but unfortunately, hypertension and hypnotic medications were not accounted for [44]. A smaller study of 189 individuals interestingly showed significantly lower MoCA scores with long sleep duration, but not MMSE scores [45]. In contrast with these studies however, worse MMSE scores [59, 60] and immediate and delayed recall [60] have also been associated with shorter sleep. One study showed a linear relationship of worsening global cognition over 2 years with every hour of sleep less than 7 h, when adjusted for sex, age, education, and BMI [61]. Therefore, the broad nature of these cognitive tests may have contributed to different findings.

Secondly, different definitions and thresholds of sleep durations have been applied across studies as ‘long’ sleep duration can range from greater than 7 h to greater than 11 h [42–45, 47, 48]. Conversely, a ‘short’ sleep duration can range from less than 8 h to less than 4 h depending on the specific study [56, 59–63], which may also contribute to the heterogeneity of results.

**Fig. 1:**
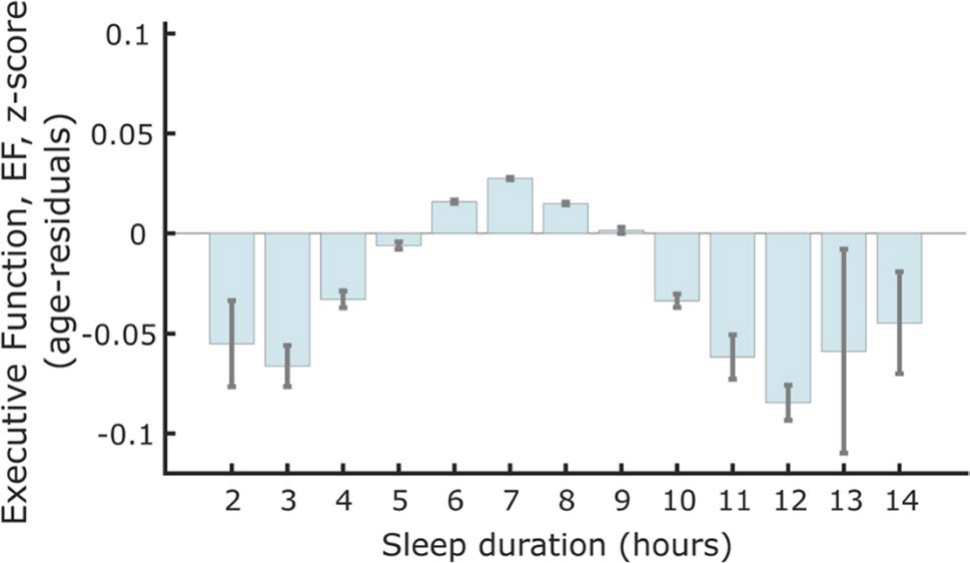
Association between sleep duration and standardised executive function score from a study of 474,417 individuals in the UK Biobank. Seven hours of self-reported sleep duration was associated with the highest executive function score. A negative relationship was present with sleeping less than 6 h and more sleep from 8 h (Tai et al. 2022, reproduced with permission from the author)

Sleep thresholds are now less important, as recent studies have been able to investigate how every hour of sleep reported relates to executive function. This has been made possible by large study cohorts which have consistently identified a quadratic, or inverted ‘U’-shaped, relationship between self-reported sleep duration and executive function with increasingly worse performance with both less and more sleep around a baseline of 7–8 h [49–58, 64, 65]. A study of around 480,000 individuals, aged 38–73 years, showed that 7 h of sleep per day was associated with the highest executive function performance, using a measure derived from specific computer-based tasks of attention and working memory. Furthermore, there was a parametric decline in executive function associated with every hour of sleep below and above 7 h suggesting an optimal sleep duration (Fig. 1). This finding was consistent for individuals who were below and above the age of 60 years, suggesting that an optimal sleep duration exists as individuals age. This study also showed a similar quadratic relationship between sleep duration and brain volume across 46 different cortical regions which highlights how sleep may be important for brain health [54•].

The quadratic relationship between executive function and sleep [54, 64] is observed in other cognitive domains including memory [51, 53, 57, 64], visuospatial abilities [51, 65], verbal fluency [49, 57], and global cognitive tests like the MMSE [50, 55, 56]. This quadratic association is also observed in a large study of around 513,000 participants, aged 15–89 years old, in a non-supervised, online ‘game-based’ test of processing speed, working memory, arithmetic, and visuospatial memory [66]. These findings are additionally confirmed in a more recent meta-analysis that self-reported short and long sleep increased the odds of cognitive impairment by 1.40 and 1.58 respectively [58]. Therefore, cross-sectional studies indicate both long and shorter sleep durations may be detrimental to executive functioning and, importantly, not just the extremes of sleep deprivation and over-sleeping.

### Longitudinal Measurements of Sleep Duration Identify Similar Patterns with Executive Function

Cross-sectional studies compare executive function and sleep duration at a single time point. A limitation of this approach is the inability to infer causality. Longitudinal studies, which are more costly to run, offer more information in this regard although cannot strictly determine causality either. Several longitudinal studies have examined sleep duration and executive function. A study where both cognition and self-reported sleep were measured at 3 time points over 10 years showed that long sleep duration was associated with worse global cognition, but not specific cognitive domains. Unfortunately, this study failed to adjust for confounders such as depression, hypnotic use, and sleep apnoea [67]. Another longitudinal study used a combination of EEG measurement and self-reported sleep following 100 participants over 4 years, and showed worsening cognition over time was associated with both short and long sleep durations, similar to cross-sectional studies [68•]. A change in sleep duration out of the optimal range, over a 5-year follow-up, was also related to worse performance in MMSE, fluency, and reasoning tasks, but had no effect on memory [69]. Longitudinal studies therefore indicate that average sleep duration, in a similar pattern to cross-sectional studies, can affect executive function and cognitive ability in the future.

### Sub-optimal Sleep Duration May Predict Dementia Onset

An important consideration is whether worsening executive function over time may represent the development of dementia. One study of 2457 elderly participants from the Framingham cohort showed double the risk in those who reported long sleep to be diagnosed with dementia, even when adjusted for a genetic predisposition for Alzheimer’s. Transitioning to long sleep was also associated with a higher risk, than those who previously slept for long durations [70]. Two 2019 meta-analyses support the findings of long sleep being associated with incident dementia [71, 72]. However, a well-controlled longitudinal study of 7959 participants of the Whitehall II study over 25 years indicated that self-reported short sleep duration in mid-life was associated with incident dementia when elderly. They further confirmed this association with objective sleep measures in a subpopulation of 3888 participants. There was no link with long sleep, which they report is due to the fact they are looking at sleep durations from mid-life, whereas other studies focus on the elderly—when any impending dementia may already be affecting sleep patterns [73••]. Therefore, there is evidence that both short and long reported sleep duration may be associated with developing dementia.

### Objective Measurements of Sleep Duration and Executive Function

Why are both long and short durations associated with worse executive function? There may be biological reasons, which will be discussed below, as well as practical reasons. Self-reported sleep habits from large cross-sectional studies may not represent true sleep characteristics, as individuals may either over- or under-estimate how long they sleep. Generally, people tend to report ‘time in bed’ rather than actual time asleep [74]. There are also a tendency for those with insomnia to under-report sleep duration and a tendency of those with fragmented sleep (e.g. those with obstructive sleep apnoea (OSA) or depression) to over-report sleep duration [75, 76].

More accurate data comes from electroencephalography (EEG) or actigraphy studies, which objectively measure when an individual is sleeping and delineate sleep stages. While EEG studies are more difficult to carry out and often have smaller sample sizes, actigraphy is increasingly used in large samples to investigate sleep duration [46, 77, 78]. Results from some EEG and actigraphy studies conflict with self-reported sleep data, with total sleep time showing little association with executive function. Blackwell et al. found that total sleep time (TST) measured by actigraphy was related to MMSE score, but not to the trail-making test [46], while Suemoto et al. demonstrated no impairments related to actigraphy-measured TST (10-word list, verbal fluency, and trail-making tests) [79]. Similarly, a meta-analysis of actigraphy and EEG studies showed no associations with TST. Importantly though, early studies were limited by the use of linear analysis models, which may have missed the quadratic relationship recently described between sleep duration and performance. However, a longitudinal study that used both EEG measurement and self-reported sleep had findings consistent with cross-sectional studies with worsening cognition over time in short and long sleep durations [68•]. These objective sleep studies hint at a relationship between sleep and cognition that may go beyond just length of sleep.

In summary, recent literature has emphasised the quadratic relationship between sleep duration and executive function and suggests that there may be an optimal duration of sleep to maximise our cognitive performance. This has both personal and public health implications. However, an interesting and important question that has emerged from objective sleep monitoring studies is whether total sleep duration alone is the best measure of sleep. In the next section, we consider how sleep characteristics other than sleep duration may be relevant to the relationship with executive function.

## Sleep Quality May Be More Relevant for Long-Term Cognitive Outcomes than Absolute Duration

Sleep quality may be more important than sleep duration alone when considering the impact on executive function. Evidence from studies with objective sleep recording rather than self-reported sleep duration indicates that time spent in different sleep stages and sleep fragmentation may correlate better to cognitive function than total sleep time alone [78–83]. Subjective sleep quality, such as asking whether the participants felt well rested, also correlates better to cognitive function over absolute duration [84].

There are three main reasons for why this effect of sleep ‘quality over quantity’ in relation to cognition may be relevant. Firstly, as discussed previously, there may be several biases with self-reported sleep duration [74–76], which may mean the extremes of self-reported sleep durations are acting as a surrogate for poor sleep quality.

Secondly, conditions associated with poor sleep quality are also often related to poor general cognition. This could confound the relationship between sleep and executive function. For example, individuals with OSA can wake numerous times overnight with brief apnoeic spells resulting in poor sleep. This condition is associated with obesity and resistant hypertension [85, 86], which can lead to a decline in cognition via small vessel disease in the brain. Thirdly, sleep architecture evolves with age. Sleep efficiency (SE) (time spent asleep between first falling asleep and waking in the morning) decreases from 89 to 79% from middle age to 70 years old, and the change accelerates over the age of 70 [87]. Given there is concurrent cognitive decline naturally in this time [8–10], parsing out the effect of sleep and other factors is difficult but important.

Several studies have explored the link between sleep quality and executive function using various methods. Actigraphy uses a wearable device that measures movements when going to bed to assess parameters like total sleep time (TST), sleep onset latency (SOL), wake after sleep onset (WASO), and general restlessness to gauge quality of sleep. EEG studies tend to use time in slow wave sleep (SWS), rapid eye movement sleep (REM), and non-REM sleep (nREM), as well as the presence and density of sleep spindles [88, 89]. Other studies rely on participants reporting whether or not they had a restful and restorative sleep [83, 84]. Together, these may be used to give an indication of ‘sleep quality’, or of ‘sleep fragmentation’, rather than self-reported sleep duration.

Feeling rested after sleep or not, regardless of actual time reported asleep, was reported to be more indicative of speed and flexibility of processing [67]. Similarly, Teräs et al. reported better executive function in those who reported restorative sleep, in a cross-sectional study of healthy mid-old age participants [83]. Those with more restful nights did better in memory tasks, and those with decreased SOL did better in executive function tasks in a meta-analysis of actigraphy-measured sleep and cognition [81••]. More time in REM was associated with better executive function in adults aged 20–84 (tested with a goal neglect task), and more SWS and sleep spindles were associated with faster response times, an indication of attention and reaction time [80]. Sleep spindle density was also recently reported to be associated with better executive function (using DSST, card sorting, and Stroop) and MoCA scores in a cross-sectional study of sedentary 63 middle-older aged participants [90].

A recent prospective study investigating incident cognitive impairment 4 years after baseline polysomnography found a small, but statistically significant, association of shorter average sleep cycle length and average REM duration [82]. A similar study published in 2023 additionally reported no association on executive function or global cognitive performance with actigraphy-measured TST or SOL. They did however have small associations between lower SE and poorer visuospatial ability. A limitation of the study was only having follow-up data on 70% of the original participants. They also commented on different associations seen between the (self-identified) White and Black participants; with poor sleep having a greater effect on Black participants [91].

In summary, executive function appears to be reliably related to sleep quality as measured by sleep onset latency, wake after sleep onset, and whether participants seem rested or not. EEG studies indicate that time in REM and SWS may be important to the mechanism by which sleep affects cognition. Sleep quality should be considered and investigated specifically in any future studies investigating the link between sleep and executive function.

## Biological Mechanisms Underlying the Link Between Sleep and Executive Function

**Fig. 2:**
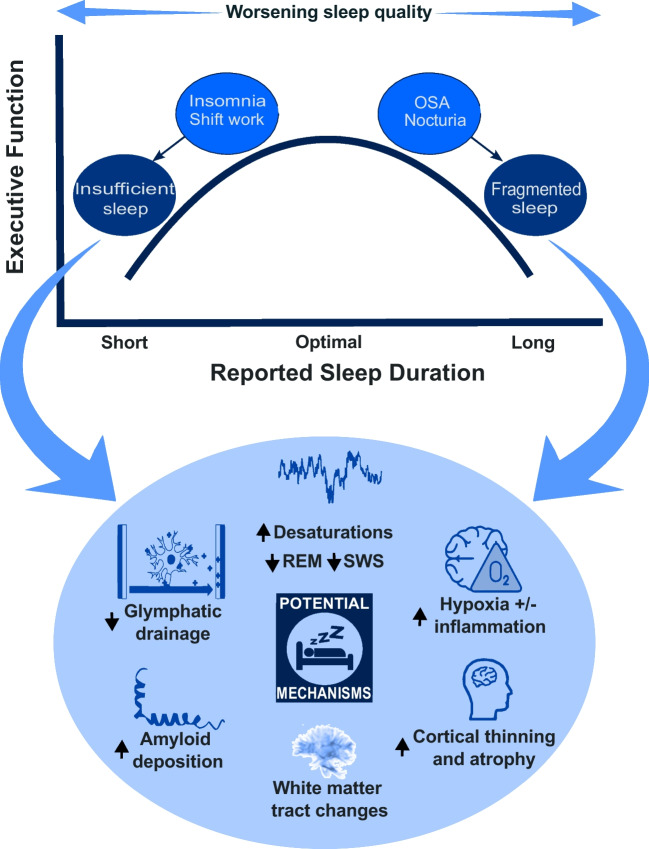
A summary diagram illustrating the relationship between self-reported sleep duration and executive function and the potential mechanisms by which this may occur. *SWS* slow wave sleep, *REM* rapid eye movement, *OSA* obstructive sleep apnoea

Why is sleep so important to executive function? Studies have explored several biological mechanisms that may underlie the link between sleep and executive function. These include potential changes in brain volume, alterations in brain connectivity, accumulation of neurodegenerative proteins, and disrupted glymphatic drainage (summarised in Fig. 2).

Poor sleep may lead to reduced brain volume which affects cognition. A large imaging study has demonstrated a quadratic relationship between sleep duration and executive function and multiple areas of reduced cortical volume [54•]. Cortical thinning was seen with reduced REM sleep [92] and in patients with severe OSA and sleep fragmentation; this was, importantly, shown to be partially reversible after 18 months of CPAP therapy [93].

Changes in brain connectivity may also underpin the effects of sleep deprivation. Diffusion tensor imaging, used to visualise white matter tracts in the brain, has demonstrated changes in structural brain connectivity after just one night of sleep deprivation [27, 94] and with prolonged sleep restriction [95, 96]. A study of young healthy volunteers indicated that goal-directed learning mainly recruited the ventro-medial prefrontal cortex (vmPFC) on fMRI; after sleep deprivation, this activation was less pronounced reflecting worse functional brain connectivity [6].

Beyond changes in brain structure, studies using positron emission tomography (PET) imaging [97] suggest accumulation of neurodegenerative proteins is associated with sleep deprivation [98]. Beta-amyloid is one of two main pathological proteins described in Alzheimer’s disease, the most common form of dementia. Increased amyloid plaques on PET scan, along with reduced cerebrospinal fluid (CSF) amyloid (indicating increased amyloid deposition) over 2 years, were described in 208 cognitively healthy elderly people with OSA [99]. Beta-amyloid plaques are also increased in cognitively intact adults who have shorter and poorer quality reported sleep, as well as those with poor objective sleep quality [100]. While not the remit of this review, there is a growing animal model literature which corroborates findings of accelerated amyloid plaque and tau tangle formation in sleep-deprived states [101].

More recently, the role of glymphatic drainage, representing the waste clearance system of the brain, has been proposed as a mechanism by which amyloid and other toxic metabolites are removed from the brain. One line of evidence suggests that amyloid production in Alzheimer’s may be the same as in healthy people, but that clearance is significantly slowed [100]. The glymphatic system is primarily active during sleep and affected by several factors including sleep architecture (more active during SWS) and the general physiological milieu including hormones like cortisol and noradrenaline [100, 102, 103]. Amyloid uptake shows an inverse relationship with nREM slow wave activity [104]. Amyloid levels in the interstitia are higher in wakefulness in mice, and a small human study found similar results [105], indicating that sleep deprivation may lead to higher amyloid plaque levels via reduced clearance from the brain. This offers a tangible mechanism linking poor sleep to worse cognitive functions and, possibly, increased risk of dementia.

In summary, there are several mechanisms in which poor sleep may contribute to impaired executive function with reduced quality of sleep. The underlying process is likely to be multifactorial involving a complex relationship between these biological processes (Fig. 2). Future studies must consider this complexity to better understand the causal nature between sleep and cognition.

## Conclusion and Future Directions

The prospect that sleep may be a modifiable lifestyle factor that can improve our executive function and reduce risk of dementia is both tantalising and real. This is important, especially considering that the worldwide prevalence of dementia is expected to increase by 117% from 2019 to 2050 [106]. There is consistent evidence for an optimal duration of sleep for cognitive function which is relevant to the personal health of every ageing individual. It is important, however, to remember that these findings reflect a group effect and the exact optimal duration may differ between individuals. Furthermore, other sleep factors may be equally important as the duration of sleep.

Future studies should consider both objective sleep duration and quality by incorporating detailed sleep measurements using actigraphy or EEG where possible. The potential benefits justify the costs of such studies at large scale, while the advent of machine learning and artificial intelligence will allow better data processing and interpretation. Development of short, pragmatic cognitive batteries [107] which can be performed remotely and are not culturally specific would improve the feasibility of large-scale, standardised multicentre studies. These technologies would allow longitudinal tracking of cognition and sleep in which, as described, very little research has been conducted to date. The causal direction between sleep and executive function should be further explored through interventional trials which may have an active arm of individuals with targeted sleep advice and support compared to match controls. Furthermore, using alternate approaches such as Mendelian randomisation to leverage genetic information [108] should be performed in larger, diverse populations.

From a scientific perspective, we must better understand the underlying mechanisms linking sleep to cognition and, especially, to the risk of dementia. Moving forward, sleep studies in humans would benefit from the arrival of plasma biomarkers of neurodegeneration such as beta-amyloid, phosphorylated tau, and neurofilament light chain [109]. Such in vivo and minimally invasive measurements of pathological processes have revolutionised the current landscape of dementia research and clinical trials. It is a clear next step for the study of sleep, cognition, and dementia.

In this review, we have tried to identify what is currently understood around sleep and executive function. We have highlighted the expansive literature around sleep duration and executive function and the growing importance of examining sleep quality. We have considered important questions around causality and underlying mechanisms while showing broadly what is currently understood. Finally, we have discussed areas of future research that may expand our understanding around sleep as a modifiable lifestyle factor for cognition, specifically executive function, and the global problem of dementia.

## Data Availability

Not applicable.

## References

[1] Tarokh L, Saletin JM, Carskadon MA (2016). Sleep in adolescence: physiology, cognition and mental health. Neurosci Biobehav Rev.

[2] Luyster FS, Strollo PJ, Zee PC, Walsh JK (2012). Sleep: a health imperative. Sleep.

[3] Depner CM, Stothard ER, Wright KP (2014). Metabolic consequences of sleep and circadian disorders. Curr Diab Rep.

[4] Swanson CM, Kohrt WM, Buxton OM, Everson CA, Wright KP, Orwoll ES, Shea SA (2018). The importance of the circadian system & sleep for bone health. Metabolism.

[5] Yeo H, Lee J, Jeon S, Lee S, Hwang Y, Kim J, Kim SJ (2022). Sleep disturbances, depressive symptoms, and cognitive efficiency as determinants of mistakes at work in shift and non-shift workers. Front Public Health.

[6] Chen J, Liang J, Lin X, Zhang Y, Zhang Y, Lu L, Shi J (2017). Sleep deprivation promotes habitual control over goal-directed control: behavioral and neuroimaging evidence. J Neurosci.

[7] Benarroch EE (2021). Neuroscience for clinicians: basic processes, circuits, disease mechanisms, and therapeutic implications.

[8] Park DC, Lautenschlager G, Hedden T, Davidson NS, Smith AD, Smith PK (2002). Models of visuospatial and verbal memory across the adult life span. Psychol Aging.

[9] Cansino S, Hernández-Ramos E, Estrada-Manilla C, Torres-Trejo F, Martínez-Galindo JG, Ayala-Hernández M, Gómez-Fernández T, Osorio D, Cedillo-Tinoco M, Garcés-Flores L, Beltrán-Palacios K, García-Lázaro HG, García-Gutiérrez F, Cadena-Arenas Y, Fernández-Apan L, Bärtschi A, Rodríguez-Ortiz MD (2013). The decline of verbal and visuospatial working memory across the adult life span. Age (Dordr).

[10] Hale S, Rose NS, Myerson J, Strube MJ, Sommers M, Tye-Murray N, Spehar B (2011). The structure of working memory abilities across the adult life span. Psychol Aging.

[11] Pohjasvaara T, Leskelä M, Vataja R, Kalska H, Ylikoski R, Hietanen M, Leppävuori A, Kaste M, Erkinjuntti T (2002). Post-stroke depression, executive dysfunction and functional outcome. Eur J Neurol.

[12] Azouvi P, Arnould A, Dromer E, Vallat-Azouvi C (2017). Neuropsychology of traumatic brain injury: an expert overview. Rev Neurol (Paris).

[13] Olney NT, Spina S, Miller BL (2017). Frontotemporal dementia. Neurol Clin.

[14] Zhang S, Shen L, Jiao B (2022). Cognitive dysfunction in repeat expansion diseases: a review. Front Aging Neurosci.

[15] Bowie CR, Harvey PD (2006). Administration and interpretation of the trail making test. Nat Protoc.

[16] Boake C (2002). From the Binet-Simon to the Wechsler-Bellevue: tracing the history of intelligence testing. J Clin Exp Neuropsychol.

[17] Lezak MD (2004). Neuropsychological Assessment.

[18] Stroop JR (1935). Studies of interference in serial verbal reactions. J Exp Psychol.

[19] Periáñez JA, Lubrini G, García-Gutiérrez A, Ríos-Lago M (2021). Construct validity of the stroop color-word test: influence of speed of visual search, verbal fluency, working memory, cognitive flexibility, and conflict monitoring. Arch Clin Neuropsychol.

[20] Folstein MF, Folstein SE, McHugh PR (1975). “Mini-mental state”. A practical method for grading the cognitive state of patients for the clinician. J Psychiatr Res.

[21] Nasreddine ZS, Phillips NA, Bédirian V, Charbonneau S, Whitehead V, Collin I, Cummings JL, Chertkow H (2005). The Montreal Cognitive Assessment, MoCA: a brief screening tool for mild cognitive impairment. J Am Geriatr Soc.

[22] Richter FR, Yeung N (2012). Memory and cognitive control in task switching. Psychol Sci.

[23] Ledochowski J, Andrade BF, Toplak ME (2019). A novel unstructured performance-based task of executive function in children with attention-deficit/hyperactivity disorder. J Clin Exp Neuropsychol.

[24] Yochim BP, Baldo JV, Kane KD, Delis DC (2009). D-KEFS Tower Test performance in patients with lateral prefrontal cortex lesions: the importance of error monitoring. J Clin Exp Neuropsychol.

[25] Liu Y, Wheaton AG, Chapman DP, Cunningham TJ, Lu H, Croft JB. Prevalence of Healthy Sleep Duration among Adults — United States, 2014. MMWR Morb Mortal Wkly Rep 2016;65:137–141. 10.15585/mmwr.mm6506a1 10.15585/mmwr.mm6506a1 26890214

[26] Williamson A, Feyer A (2000). Moderate sleep deprivation produces impairments in cognitive and motor performance equivalent to legally prescribed levels of alcohol intoxication. Occup Environ Med.

[27] Krause AJ, Ben Simon E, Mander BA, Greer SM, Saletin JM, Goldstein-Piekarski AN, Walker MP (2017). The sleep-deprived human brain. Nat Rev Neurosci.

[28] Pilcher JJ, McClelland LE, Moore DD, Haarmann H, Baron J, Wallsten TS, McCubbin JA (2007). Language performance under sustained work and sleep deprivation conditions. Aviat Space Environ Med.

[29] Pilcher JJ, Band D, Odle-Dusseau HN, Muth ER (2007). Human performance under sustained operations and acute sleep deprivation conditions: toward a model of controlled attention. Aviat Space Environ Med.

[30] Killgore WDS, Kerkhof GA, van Dongen HPA (2010). Effects of sleep deprivation on cognition. Progress in brain research.

[31] Chen J, Gong X, Wang L, Xu M, Zhong X, Peng Z, Song T, Xu L, Lian J, Shao Y, Weng X (2023). Altered postcentral connectivity after sleep deprivation correlates to impaired risk perception: a resting-state functional magnetic resonance imaging study. Brain Sci.

[32] Petrofsky LA, Heffernan CM, Gregg BT, Smith-Forbes EV (2022). Effects of sleep deprivation in military service members on cognitive performance: a systematic review. Mil Behav Health.

[33] Diekelmann S, Born J (2010). The memory function of sleep. Nat Rev Neurosci.

[34] Berres S, Erdfelder E (2021). The sleep benefit in episodic memory: an integrative review and a meta-analysis. Psychol Bull.

[35] Stepan ME, Fenn KM, Altmann EM (2019). Effects of sleep deprivation on procedural errors. J Exp Psychol Gen.

[36] Kurniawan IT, Cousins JN, Chong PLH, Chee MWL (2016). Procedural performance following sleep deprivation remains impaired despite extended practice and an afternoon nap. Sci Rep.

[37] Newbury CR, Crowley R, Rastle K, Tamminen J (2021). Sleep deprivation and memory: meta-analytic reviews of studies on sleep deprivation before and after learning. Psychol Bull.

[38] Arnal PJ, Sauvet F, Leger D, van Beers P, Bayon V, Bougard C, Rabat A, Millet GY, Chennaoui M (2015). Benefits of sleep extension on sustained attention and sleep pressure before and during total sleep deprivation and recovery. Sleep.

[39] Scullin MK, Bliwise DL (2015). Sleep, cognition, and normal aging: integrating a half-century of multidisciplinary research. Perspect Psychol Sci.

[40] Spencer RMC, Gouw AM, Ivry RB (2007). Age-related decline of sleep-dependent consolidation. Learn Mem.

[41] Aly M, Moscovitch M (2010). The effects of sleep on episodic memory in older and younger adults. Memory.

[42] Faubel R, López-García E, Guallar-Castillón P, Graciani A, Banegas JR, Rodríguez-Artalejo F (2009). Usual sleep duration and cognitive function in older adults in Spain. J Sleep Res.

[43] Kondo R, Miyano I, Lee S, Shimada H, Kitaoka H (2021). Association between self-reported night sleep duration and cognitive function among older adults with intact global cognition. Int J Geriatr Psychiatry.

[44] Low DV, Wu MN, Spira AP (2019). Sleep duration and cognition in a nationally representative sample of U.S. older adults. Am J Geriatr Psychiatry.

[45] Malek-Ahmadi M, Kora K, O’Connor K, Schofield S, Coon D, Nieri W (2016). Longer self-reported sleep duration is associated with decreased performance on the montreal cognitive assessment in older adults. Aging Clin Exp Res.

[46] Blackwell T, Yaffe K, Ancoli-Israel S, Schneider JL, Cauley JA, Hillier TA, Fink HA, Stone KL, Study of Osteoporotic Fractures Group (2006). Poor sleep is associated with impaired cognitive function in older women: the study of osteoporotic fractures. J Gerontol A Biol Sci Med Sci.

[47] Benito-León J, Louis ED, Bermejo-Pareja F (2013). Cognitive decline in short and long sleepers: a prospective population-based study (NEDICES). J Psychiatr Res.

[48] Kim H-B, Myung S-K, Lee S-M, Park YC, Group TKM-A (KORMA) S (2016). Longer duration of sleep and risk of cognitive decline: a meta-analysis of observational studies. NED.

[49] Ma Y, Liang L, Zheng F, Shi L, Zhong B, Xie W (2020). Association between sleep duration and cognitive decline. JAMA Netw Open.

[50] Ramos AR, Dong C, Elkind MSV, Boden-Albala B, Sacco RL, Rundek T, Wright CB (2013). Association between sleep duration and the mini-mental score: the Northern Manhattan study. J Clin Sleep Med.

[51] Li M, Wang N, Dupre ME (2022). Association between the self-reported duration and quality of sleep and cognitive function among middle-aged and older adults in China. J Affect Disord.

[52] Kyle SD, Sexton CE, Feige B, Luik AI, Lane J, Saxena R, Anderson SG, Bechtold DA, Dixon W, Little MA, Ray D, Riemann D, Espie CA, Rutter MK, Spiegelhalder K (2017). Sleep and cognitive performance: cross-sectional associations in the UK Biobank. Sleep Med.

[53] Xu L, Jiang CQ, Lam TH, Liu B, Jin YL, Zhu T, Zhang WS, Cheng KK, Thomas GN (2011). Short or long sleep duration is associated with memory impairment in older chinese: the Guangzhou Biobank Cohort Study. Sleep.

[54] Tai XY, Chen C, Manohar S, Husain M (2022). Impact of sleep duration on executive function and brain structure. Commun Biol.

[55] Chen W-C, Wang X-Y (2022). Longitudinal associations between sleep duration and cognitive impairment in Chinese elderly. Front Aging Neurosci.

[56] Devore EE, Grodstein F, Schernhammer ES (2016). Sleep duration in relation to cognitive function among older adults: a systematic review of observational studiNeuroepidemiology.

[57] Kronholm E, Sallinen M, Suutama T, Sulkava R, Era P, Partonen T (2009). Self-reported sleep duration and cognitive functioning in the general population. J Sleep Res.

[58] Lo JC, Groeger JA, Cheng GH, Dijk D-J, Chee MWL (2016). Self-reported sleep duration and cognitive performance in older adults: a systematic review and meta-analysis. Sleep Med.

[59] Deng X, Pan X, Cheng X, Zhang J, Wang L, Sang S, Zhong C, Fei G (2022). Sleep duration positively correlates with global cognition in the non-demented older adults with high school or above education. J Integr Neurosci.

[60] Winer JR, Deters KD, Kennedy G, Jin M, Goldstein-Piekarski A, Poston KL, Mormino EC (2021). Association of short and long sleep duration with amyloid-β burden and cognition in aging. JAMA Neurol.

[61] Lo JC, Loh KK, Zheng H, Sim SKY, Chee MWL (2014). Sleep duration and age-related changes in brain structure and cognitive performance. Sleep.

[62] Tworoger SS, Lee S, Schernhammer ES, Grodstein F (2006). The association of self-reported sleep duration, difficulty sleeping, and snoring with cognitive function in older women. Alzheimer Dis Assoc Disord.

[63] Ohayon MM, Vecchierini M-F (2002). Daytime sleepiness and cognitive impairment in the elderly population. Arch Intern Med.

[64] Ramos AR, Tarraf W, Daviglus M, Davis S, Gallo LC, Mossavar-Rahmani Y, Penedo FJ, Redline S, Rundek T, Sacco RL, Sotres-Alvarez D, Wright CB, Zee PC, González HM (2016). Sleep duration and neurocognitive function in the hispanic community health study/study of Latinos. Sleep.

[65] Wild CJ, Nichols ES, Battista ME, Stojanoski B, Owen AM (2018). Dissociable effects of self-reported daily sleep duration on high-level cognitive abilities. Sleep.

[66] Richards A, Inslicht SS, Metzler TJ, Mohlenhoff BS, Rao MN, O’Donovan A, Neylan TC (2017). Sleep and cognitive performance from teens to old age: more is not better. Sleep.

[67] van Oostrom SH, Nooyens ACJ, van Boxtel MPJ, Verschuren WMM (2018). Long sleep duration is associated with lower cognitive function among middle-age adults - the Doetinchem Cohort Study. Sleep Med.

[68] Lucey BP, Wisch J, Boerwinkle AH, Landsness EC, Toedebusch CD, McLeland JS, Butt OH, Hassenstab J, Morris JC, Ances BM, Holtzman DM (2021). Sleep and longitudinal cognitive performance in preclinical and early symptomatic Alzheimer’s disease. Brain.

[69] Ferrie JE, Shipley MJ, Akbaraly TN, Marmot MG, Kivimäki M, Singh-Manoux A (2011). Change in sleep duration and cognitive function: findings from the Whitehall II Study. Sleep.

[70] Westwood AJ, Beiser A, Jain N, Himali JJ, DeCarli C, Auerbach SH, Pase MP, Seshadri S (2017). Prolonged sleep duration as a marker of early neurodegeneration predicting incident dementia. Neurology.

[71] Fan L, Xu W, Cai Y, Hu Y, Wu C (2019). Sleep duration and the risk of dementia: a systematic review and meta-analysis of prospective cohort studies. J Am Med Dir Assoc.

[72] Liang Y, Qu L-B, Liu H (2019). Non-linear associations between sleep duration and the risks of mild cognitive impairment/dementia and cognitive decline: a dose-response meta-analysis of observational studies. Aging Clin Exp Res.

[73] Sabia S, Fayosse A, Dumurgier J, van Hees VT, Paquet C, Sommerlad A, Kivimäki M, Dugravot A, Singh-Manoux A (2021). Association of sleep duration in middle and old age with incidence of dementia. Nat Commun.

[74] Lauderdale DS, Knutson KL, Yan LL, Liu K, Rathouz PJ (2008). Sleep duration: how well do self-reports reflect objective measures? The CARDIA Sleep Study. Epidemiology.

[75] Trimmel K, Eder HG, Böck M, Stefanic-Kejik A, Klösch G, Seidel S (2021). The (mis)perception of sleep: factors influencing the discrepancy between self-reported and objective sleep parameters. J Clin Sleep Med.

[76] Scarlett S, Nolan HN, Kenny RA, O’Connell MDL (2021). Discrepancies in self-reported and actigraphy-based sleep duration are associated with self-reported insomnia symptoms in community-dwelling older adults. Sleep Health.

[77] Luik AI, Zuurbier LA, Hofman A, Van Someren EJW, Ikram MA, Tiemeier H (2015). Associations of the 24-h activity rhythm and sleep with cognition: a population-based study of middle-aged and elderly persons. Sleep Med.

[78] Blackwell T, Yaffe K, Laffan A, Ancoli-Israel S, Redline S, Ensrud KE, Song Y, Stone KL, Osteoporotic Fractures in Men (MrOS) Study Group (2014). Associations of objectively and subjectively measured sleep quality with subsequent cognitive decline in older community-dwelling men: the MrOS sleep study. Sleep.

[79] Suemoto CK, Santos RB, Giatti S, Aielo AN, Silva WA, Parise BK, Cunha LF, Souza SP, Griep RH, Brunoni AR, Lotufo PA, Bensenor IM, Drager LF (2023). Association between objective sleep measures and cognitive performance: a cross-sectional analysis in the Brazilian Longitudinal Study of Adult Health (ELSA-Brasil) study. J Sleep Res.

[80] Della Monica C, Johnsen S, Atzori G, Groeger JA, Dijk D-J (2018). Rapid eye movement sleep, sleep continuity and slow wave sleep as predictors of cognition, mood, and subjective sleep quality in healthy men and women, aged 20–84 years. Front Psychiatry.

[81] Qin S, Leong RLF, Ong JL, Chee MWL (2022). Associations between objectively measured sleep parameters and cognition in healthy older adults: a meta-analysis. Sleep Med Rev.

[82] Suh SW, Han JW, Lee JR, Byun S, Kwak KP, Kim BJ, Kim SG, Kim JL, Kim TH, Ryu S-H, Moon SW, Park JH, Seo J, Youn JC, Lee DY, Lee DW, Lee SB, Lee JJ, Jhoo JH, Yoon IY, Kim KW (2019). Short average duration of NREM/REM cycle is related to cognitive decline in an elderly cohort: an exploratory investigation. J Alzheimers Dis.

[83] Teräs T, Rovio S, Spira AP, Myllyntausta S, Pulakka A, Vahtera J, Stenholm S (2020). Associations of accelerometer-based sleep duration and self-reported sleep difficulties with cognitive function in late mid-life: the Finnish Retirement and Aging Study. Sleep Med.

[84] Zavecz Z, Nagy T, Galkó A, Nemeth D, Janacsek K (2020). The relationship between subjective sleep quality and cognitive performance in healthy young adults: evidence from three empirical studies. Sci Rep.

[85] Lombardi C, Tobaldini E, Montano N, Losurdo A, Parati G (2017). Obstructive sleep apnea syndrome (OSAS) and cardiovascular system. Med Lav.

[86] O’Donnell C, O’Mahony AM, McNicholas WT, Ryan S (2021). Cardiovascular manifestations in obstructive sleep apnea: current evidence and potential mechanisms. Pol Arch Intern Med.

[87] Pace-Schott EF, Spencer RMC (2011). Age-related changes in the cognitive function of sleep. Prog Brain Res.

[88] Marino M, Li Y, Rueschman MN, Winkelman JW, Ellenbogen JM, Solet JM, Dulin H, Berkman LF, Buxton OM (2013). Measuring sleep: accuracy, sensitivity, and specificity of wrist actigraphy compared to polysomnography. Sleep.

[89] Walia HK, Mehra R, Levin KH, Chauvel P (2019). Chapter 24 - Practical aspects of actigraphy and approaches in clinical and research domains. Handbook of Clinical Neurology.

[90] Guadagni V, Byles H, Tyndall AV, Parboosingh J, Longman RS, Hogan DB, Hanly PJ, Younes M, Poulin MJ (2021). Association of sleep spindle characteristics with executive functioning in healthy sedentary middle-aged and older adults. J Sleep Res.

[91] Owusu JT, Rabinowitz JA, Tzuang M, An Y, Kitner-Triolo M, Zipunnikov V, Wu MN, Wanigatunga SK, Schrack JA, Thorpe RJ, Simonsick EM, Ferrucci L, Resnick SM, Spira AP (2023). Associations between objectively measured sleep and cognition: main effects and interactions with race in adults aged ≥50 years. J Gerontol: Series A.

[92] Sanchez-Espinosa MP, Atienza M, Cantero JL (2014). Sleep deficits in mild cognitive impairment are related to increased levels of plasma amyloid-β and cortical thinning. Neuroimage.

[93] Kim H, Joo E, Suh S, Kim J-H, Kim ST, Hong SB (2016). Effects of long-term treatment on brain volume in patients with obstructive sleep apnea syndrome. Hum Brain Mapp.

[94] Voldsbekk I, Groote I, Zak N, Roelfs D, Geier O, Due-Tønnessen P, Løkken L-L, Strømstad M, Blakstvedt TY, Kuiper YS, Elvsåshagen T, Westlye LT, Bjørnerud A, Maximov II (2021). Sleep and sleep deprivation differentially alter white matter microstructure: a mixed model design utilising advanced diffusion modelling. Neuroimage.

[95] Khalsa S, Hale JR, Goldstone A, Wilson RS, Mayhew SD, Bagary M, Bagshaw AP (2017). Habitual sleep durations and subjective sleep quality predict white matter differences in the human brain. Neurobiol Sleep Circadian Rhythms.

[96] Lee M-H, Lee Y, Hwang YH, Min A, Han BS, Kim DY (2016). Impact of sleep restriction on the structural brain network. NeuroReport.

[97] Kolanko MA, Win Z, Loreto F, Patel N, Carswell C, Gontsarova A, Perry RJ, Malhotra PA (2020). Amyloid PET imaging in clinical practice. Pract Neurol.

[98] Shokri-Kojori E, Wang G-J, Wiers CE, Demiral SB, Guo M, Kim SW, Lindgren E, Ramirez V, Zehra A, Freeman C, Miller G, Manza P, Srivastava T, De Santi S, Tomasi D, Benveniste H, Volkow ND (2018). β-Amyloid accumulation in the human brain after one night of sleep deprivation. Proc Natl Acad Sci U S A.

[99] Sharma RA, Varga AW, Bubu OM, Pirraglia E, Kam K, Parekh A, Wohlleber M, Miller MD, Andrade A, Lewis C, Tweardy S, Buj M, Yau PL, Sadda R, Mosconi L, Li Y, Butler T, Glodzik L, Fieremans E, Babb JS, Blennow K, Zetterberg H, Lu SE, Badia SG, Romero S, Rosenzweig I, Gosselin N, Jean-Louis G, Rapoport DM, de Leon MJ, Ayappa I, Osorio RS (2018). Obstructive sleep apnea severity affects amyloid burden in cognitively normal elderly. a longitudinal study. Am J Respir Crit Care Med.

[100] Boespflug EL, Iliff JJ (2018). The emerging relationship between interstitial fluid–cerebrospinal fluid exchange, amyloid-β, and sleep. Biol Psychiat.

[101] Di Meco A, Joshi YB, Praticò D (2014). Sleep deprivation impairs memory, tau metabolism, and synaptic integrity of a mouse model of Alzheimer’s disease with plaques and tangles. Neurobiol Aging.

[102] Chong PLH, Garic D, Shen MD, Lundgaard I, Schwichtenberg AJ (2022). Sleep, cerebrospinal fluid, and the glymphatic system: a systematic review. Sleep Med Rev.

[103] Xie L, Kang H, Xu Q, Chen MJ, Liao Y, Thiyagarajan M, O’Donnell J, Christensen DJ, Nicholson C, Iliff JJ, Takano T, Deane R, Nedergaard M (2013). Sleep drives metabolite clearance from the adult brain. Science.

[104] Ward SA, Pase MP (2020). Advances in pathophysiology and neuroimaging: implications for sleep and dementia. Respirology.

[105] Kang J-E, Lim MM, Bateman RJ, Lee JJ, Smyth LP, Cirrito JR, Fujiki N, Nishino S, Holtzman DM (2009). Amyloid-beta dynamics are regulated by orexin and the sleep-wake cycle. Science.

[106] Nichols E, Steinmetz JD, Vollset SE, Fukutaki K, Chalek J, Abd-Allah F, Abdoli A, Abualhasan A, Abu-Gharbieh E, Akram TT, Hamad HA, Alahdab F, Alanezi FM, Alipour V, Almustanyir S, Amu H, Ansari I, Arabloo J, Ashraf T, Astell-Burt T, Ayano G, Ayuso-Mateos JL, Baig AA, Barnett A, Barrow A, Baune BT, Béjot Y, Bezabhe WMM, Bezabih YM, Bhagavathula AS, Bhaskar S, Bhattacharyya K, Bijani A, Biswas A, Bolla SR, Boloor A, Brayne C, Brenner H, Burkart K, Burns RA, Cámera LA, Cao C, Carvalho F, Castro-de-Araujo LFS, Catalá-López F, Cerin E, Chavan PP, Cherbuin N, Chu D-T, Costa VM, Couto RAS, Dadras O, Dai X, Dandona L, Dandona R, la Cruz-Góngora VD, Dhamnetiya D, da Silva DD, Diaz D, Douiri A, Edvardsson D, Ekholuenetale M, Sayed IE, El-Jaafary SI, Eskandari K, Eskandarieh S, Esmaeilnejad S, Fares J, Faro A, Farooque U, Feigin VL, Feng X, Fereshtehnejad S-M, Fernandes E, Ferrara P, Filip I, Fillit H, Fischer F, Gaidhane S, Galluzzo L, Ghashghaee A, Ghith N, Gialluisi A, Gilani SA, Glavan I-R, Gnedovskaya EV, Golechha M, Gupta R, Gupta VB, Gupta VK, Haider MR, Hall BJ, Hamidi S, Hanif A, Hankey GJ, Haque S, Hartono RK, Hasaballah AI, Hasan MT, Hassan A, Hay SI, Hayat K, Hegazy MI, Heidari G, Heidari-Soureshjani R, Herteliu C, Househ M, Hussain R, Hwang B-F, Iacoviello L, Iavicoli I, Ilesanmi OS, Ilic IM, Ilic MD, Irvani SSN, Iso H, Iwagami M, Jabbarinejad R, Jacob L, Jain V, Jayapal SK, Jayawardena R, Jha RP, Jonas JB, Joseph N, Kalani R, Kandel A, Kandel H, Karch A, Kasa AS, Kassie GM, Keshavarz P, Khan MA, Khatib MN, Khoja TAM, Khubchandani J, Kim MS, Kim YJ, Kisa A, Kisa S, Kivimäki M, Koroshetz WJ, Koyanagi A, Kumar GA, Kumar M, Lak HM, Leonardi M, Li B, Lim SS, Liu X, Liu Y, Logroscino G, Lorkowski S, Lucchetti G, Saute RL, Magnani FG, Malik AA, Massano J, Mehndiratta MM, Menezes RG, Meretoja A, Mohajer B, Ibrahim NM, Mohammad Y, Mohammed A, Mokdad AH, Mondello S, Moni MAA, Moniruzzaman M, Mossie TB, Nagel G, Naveed M, Nayak VC, Kandel SN, Nguyen TH, Oancea B, Otstavnov N, Otstavnov SS, Owolabi MO, Panda-Jonas S, Kan FP, Pasovic M, Patel UK, Pathak M, Peres MFP, Perianayagam A, Peterson CB, Phillips MR, Pinheiro M, Piradov MA, Pond CD, Potashman MH, Pottoo FH, Prada SI, Radfar A, Raggi A, Rahim F, Rahman M, Ram P, Ranasinghe P, Rawaf DL, Rawaf S, Rezaei N, Rezapour A, Robinson SR, Romoli M, Roshandel G, Sahathevan R, Sahebkar A, Sahraian MA, Sathian B, Sattin D, Sawhney M, Saylan M, Schiavolin S, Seylani A, Sha F, Shaikh MA, Shaji KS, Shannawaz M, Shetty JK, Shigematsu M, Shin JI, Shiri R, Silva DAS, Silva JP, Silva R, Singh JA, Skryabin VY, Skryabina AA, Smith AE, Soshnikov S, Spurlock EE, Stein DJ, Sun J, Tabarés-Seisdedos R, Thakur B, Timalsina B, Tovani-Palone MR, Tran BX, Tsegaye GW, Tahbaz SV, Valdez PR, Venketasubramanian N, Vlassov V, Vu GT, Vu LG, Wang Y-P, Wimo A, Winkler AS, Yadav L, Jabbari SHY, Yamagishi K, Yang L, Yano Y, Yonemoto N, Yu C, Yunusa I, Zadey S, Zastrozhin MS, Zastrozhina A, Zhang Z-J, Murray CJL, Vos T (2022). Estimation of the global prevalence of dementia in 2019 and forecasted prevalence in 2050: an analysis for the Global Burden of Disease Study 2019. The Lancet Public Health.

[107] Geddes MR, O’Connell ME, Fisk JD, Gauthier S, Camicioli R, Ismail Z (2020). Remote cognitive and behavioral assessment: report of the Alzheimer Society of Canada Task Force on dementia care best practices for COVID-19. Alzheimers Dement (Amst).

[108] Henry A, Katsoulis M, Masi S, Fatemifar G, Denaxas S, Acosta D, Garfield V, Dale CE (2019). The relationship between sleep duration, cognition and dementia: a Mendelian randomization study. Int J Epidemiol.

[109] Cullen NC, Leuzy A, Janelidze S, Palmqvist S, Svenningsson AL, Stomrud E, Dage JL, Mattsson-Carlgren N, Hansson O (2021). Plasma biomarkers of Alzheimer’s disease improve prediction of cognitive decline in cognitively unimpaired elderly populations. Nat Commun.

